# Dynamic modelling of an *ACADS* genotype in fatty acid oxidation – Application of cellular models for the analysis of common genetic variants

**DOI:** 10.1371/journal.pone.0216110

**Published:** 2019-05-23

**Authors:** Kerstin Matejka, Ferdinand Stückler, Michael Salomon, Regina Ensenauer, Eva Reischl, Lena Hoerburger, Harald Grallert, Gabi Kastenmüller, Annette Peters, Hannelore Daniel, Jan Krumsiek, Fabian J. Theis, Hans Hauner, Helmut Laumen

**Affiliations:** 1 Chair of Nutritional Medicine, Else Kröner-Fresenius-Center for Nutritional Medicine, TUM School of Life Sciences Weihenstephan, Technische Universität München, Freising-Weihenstephan, Germany; 2 ZIEL-Research Center for Nutrition and Food Sciences, TUM School of Life Sciences Weihenstephan, Technische Universität München, Freising-Weihenstephan, Germany; 3 German Center for Diabetes Research (DZD), Neuherberg, Germany; 4 Clinical Cooperation Group Nutrigenomics and Type 2 Diabetes, Helmholtz Zentrum München, Neuherberg, Germany; 5 Clinical Cooperation Group Nutrigenomics and Type 2 Diabetes, Technische Universität München, Freising-Weihenstephan, Germany; 6 Institute of Computational Biology, Helmholtz Zentrum München, Neuherberg, Germany; 7 SIRION Biotech GmbH, Martinsried, Germany; 8 Research Center, Dr. von Hauner Children’s Hospital, Ludwig-Maximilians-Universität München, München, Germany; 9 Experimental Pediatrics and Metabolism, Department of General Pediatrics, Neonatology and Pediatric Cardiology, University Children’s Hospital, Heinrich Heine University Düsseldorf, Düsseldorf, Germany; 10 Institute of Child Nutrition, Max Rubner-Institut, Karlsruhe, Germany; 11 Research Unit of Molecular Epidemiology, Helmholtz Zentrum München, Neuherberg, Germany; 12 Institute of Epidemiology, Helmholtz Zentrum München, Neuherberg, Germany; 13 Paediatric Nutritional Medicine, Else Kröner-Fresenius-Centre for Nutritional Medicine, TUM School of Life Sciences Weihenstephan, Technische Universität München, Freising-Weihenstephan, Germany; 14 Institute of Bioinformatics and Systems Biology, Helmholtz Zentrum München, German Research Center for Environmental Health, Neuherberg, Germany; 15 German Research Center for Cardiovascular Disease (DZHK-Munich partner site), Neuherberg, Germany; 16 Chair of Physiology of Human Nutrition, TUM School of Life Sciences Weihenstephan, Technische Universität München, Freising-Weihenstephan, Germany; 17 Institute for Computational Biomedicine, Englander Institute for Precision Medicine, Department of Physiology and Biophysics, Weill Cornell Medicine, New York, United States of America; 18 Department of Mathematical Science, Technische Universität München, Garching, Germany; 19 Else Kröner-Fresenius-Center for Nutritional Medicine, Klinikum rechts der Isar, Technische Universität München, München, Germany; 20 Institute of Experimental Genetics, Helmholtz Zentrum München, Neuherberg, Germany; 21 Research Unit Protein Science, Helmholtz Zentrum München, Neuherberg, Germany; Duke University, UNITED STATES

## Abstract

**Background:**

Genome-wide association studies of common diseases or metabolite quantitative traits often identify common variants of small effect size, which may contribute to phenotypes by modulation of gene expression. Thus, there is growing demand for cellular models enabling to assess the impact of gene regulatory variants with moderate effects on gene expression. Mitochondrial fatty acid oxidation is an important energy metabolism pathway. Common noncoding acyl-CoA dehydrogenase short chain (*ACADS*) gene variants are associated with plasma C4-acylcarnitine levels and allele-specific modulation of *ACADS* expression may contribute to the observed phenotype.

**Methods and findings:**

We assessed *ACADS* expression and intracellular acylcarnitine levels in human lymphoblastoid cell lines (LCL) genotyped for a common *ACADS* variant associated with plasma C4-acylcarnitine and found a significant genotype-dependent decrease of ACADS mRNA and protein. Next, we modelled gradual decrease of ACADS expression using a tetracycline-regulated shRNA-knockdown of ACADS in Huh7 hepatocytes, a cell line with high fatty acid oxidation-(FAO)-capacity. Assessing acylcarnitine flux in both models, we found increased C4-acylcarnitine levels with decreased ACADS expression levels. Moreover, assessing time-dependent changes of acylcarnitine levels in shRNA-hepatocytes with altered ACADS expression levels revealed an unexpected effect on long- and medium-chain fatty acid intermediates.

**Conclusions:**

Both, genotyped LCL and regulated shRNA-knockdown are valuable tools to model moderate, gradual gene-regulatory effects of common variants on cellular phenotypes. Decreasing ACADS expression levels modulate short and surprisingly also long/medium chain acylcarnitines, and may contribute to increased plasma acylcarnitine levels.

## Introduction

Genome-wide association studies (GWAS) identified thousands of variants associated with diverse diseases [1]. Although inborn errors of metabolism provided numerous examples how genetics associates with metabolic traits [2], the mechanistic impact of common gene variants, often resulting from a mixture of related processes such as environmental exposures and identified loci [1,3,4], remains challenging. Expression and metabolic quantitative trait loci (eQTL, mQTL) can assist the identification of the underlying biological mechanisms that link a genotype to a phenotype, but this work requires proper cell models with the observed genetic background. However, the availability of human cell models for elucidating the functional role of common gene variants in human disease is limited [5].

Lymphoblastoid cell lines (LCL) are easily accessible in humans and frequently used and stored in biobanks as a choice of the patients’ genetic material [6–9]. Here we tested if genotyped LCLs are a valuable tool to assess allele-dependent differences in metabolic pathways. We chose an association of C4-acylcarnitine plasma levels, the transport form of the mitochondrial fatty acid oxidation (FAO) product butyric acid, with the noncoding rs2014355T>C variant located in close proximity to the short chain acyl-CoA dehydrogenase (ACADS) gene locus [10–14] and in perfect linkage disequilibrium with the non-synonymous single nucleotide polymorphism (SNP) rs1799958G>A (c.625G>A, p.G209S). The gene encodes for an enzyme catalysing mainly the conversion of butyryl (C4)-CoA to acetyl (C2)-CoA in mitochondrial FAO [15]. To assess genotype-specific effects on FAO, we adopted an *in situ* assay of oleic acid oxidation [16] for use in donor LCLs. Moreover, we generated a tet-regulated ACADS knockdown in a human hepatocyte cell line, enabling assessment of dose-dependent effects of *ACADS* mRNA and protein expression levels in a cell type with known high FAO capacity. This *in vitro* model also allowed studying the effect of ACADS expression levels on the intra- and extracellular acylcarnitine concentrations as a surrogate of FAO activity.

## Materials and methods

### Cell culture methods

LCLs were grown in RPMI medium 1640 (Gibco, Karlsruhe, Germany) supplemented with 10% fetal bovine serum (FBS) (Sigma-Aldrich, Steinheim, Germany) and cultured in uncoated 75 cm^2^ flasks at 37°C and 5% CO_2_. Cells were subcultured every 2–4 days, and cell concentrations never exceeded 1.2 x 10^6^ cells/mL. The human hepatoma cell line Huh7 [17] was cultivated in DMEM medium (Gibco, Karlsruhe; Germany) containing 10% FBS (PAA Laboratories GmbH, Pasching, Austria) and 1% penicillin-streptomycin (PAA Laboratories GmbH, Pasching, Germany) at 37°C in a humidified atmosphere at 5% CO_2_. Cells were passaged twice a week.

### Genotyping and sequencing of LCL cells

LCL immortalised with Epstein-Barr virus from three homozygous major and five homozygous minor allele carriers of the rs2014355T>C variant (age 55.5 ± 6.9 years, 6 female and 2 male donors) were obtained from the KORA cohort (Cooperative Health Research in the Augsburg Region), a population-based cohort of healthy adults from the Augsburg region in Southern Germany [18]. The Ethical Committee of the Bayerische Landesärztekammer has approved the study (#05004, 2^nd^ August 2005) and written informed consent was obtained. First, genotypes for the noncoding rs2014355 and the coding rs1799958 (NM_000017.2:c.625G>A; NP_000008.1:p.G209S) variants were verified by Sanger sequencing on an ABI3730 instrument (Applied Biosystems, Darmstadt, Germany) using standard protocols after PCR amplification of the SNP containing DNA fragment with genomic DNA as template (PCR primers: rs2014355-forward TGTCCTTAGGGTGACAGG; -reverse TCCTGCATCACTGCCGTT; rs1799958-forward TGGGCTGCTGTCATTTCT; -reverse AGTCCTCAAAGATGAGGTT). Additional sequencing of all *ACADS* exons 1–10 confirmed rs2014355 and rs1799958 genotypes and identified the synonymous variants rs3914 (c.321T>C; p.R107R) and rs3915 (c.990C>T; p.R330R). In all analysed LCLs these variants were found to be in perfect linkage disequilibrium. Moreover, sequencing confirmed the absence of any other mutation in the *ACADS* coding region in the here analysed LCL cells.

### Plasmid construction

The optimal shRNA sequence for the ACADS knockdown system was identified using the pVal shRNA Validation Platform RNAiONE (Sirion Biotech, Martinsried, Germany, http://www.sirion-biotech.com/pval_shrna_validation_platform.html,). In brief, a panel of 10 bioinformatically selected shRNA sequences with high predicted on-target activity was evaluated for activity in a cell model using the RNAiONE shRNA selection vector pVal. The best shRNA sequence showing 91% knockdown activity was used in subsequent studies. Both, shRNA revealing the best knockdown efficiency and a non-target shRNA (CAACAAGATGAAGAGCACCAA) were cloned into lentivirus inducible one-vector shmir platform (Sirion Biotech, Martinsried, Germany). Stable Huh7 cell pools (shACADS Huh7 and shNTC Huh7) were generated by packaging, transduction and stable integration of the tet-on expression vector and subsequent antibiotic selection by Sirion Biotech.

### Doxycycline treatment

To achieve different levels of ACADS expression, stable Huh7 cells transduced with the tet-inducible shRNA for down-regulation of ACADS (shACADS) or control shRNA (shNTC) were incubated with 10 ng/mL doxycycline (dox, Sigma-Aldrich, Steinheim, Germany) for 5 days and with 5 ng/mL dox for 3 days, and the medium was changed after 3 days. Knockdown efficiency was examined on RNA and protein level using qRT-PCR and western blot analysis, respectively.

### Quantitative RT-PCR

Total RNA from LCLs and Huh7 was isolated using the NucleoSpin Kit (Macherey-Nagel, Dueren, Germany) according to the manufacturer’s instructions. The reverse transcription of 300 ng LCL RNA and 1 μg Huh7 RNA into cDNA for each sample was performed using the High capacity cDNA reverse transcription kit according to the manufacturers’ protocol (high capacity cDNA reverse transcription kit, Applied Biosystems, Darmstadt, Germany). All temperature steps of the reverse transcription were performed in the Multicycler gradient ep from Eppendorf (Hamburg, Germany). PCR amplification of the human transcripts was performed using quantitative PCR Maxima SYBR-Green (Fermentas, Thermo Fisher Scientific Inc., Rockford, IL) in duplicates using the Mastercycler ep realplex from Eppendorf (Hamburg, Germany) with an initial activation of 10 min at 95°C followed by 40 cycles of 15 secs at 95°C and 40 secs at 61°C. The results were corrected for reference genes Glyceraldehyde-3-phosphate-Dehydrogenase (*GAPDH*), Tyrosine 3-Monooxygenase/Tryptophan 5-Monooxygenase Activation Protein Zeta (*YWHAZ*) and Peptidylprolyl Isomerase A (*PPIA*). The following primers (MWG Biotech, München, Germany) were designed using NCBI primer blast software (http://www.ncbi.nlm.nih.gov/tools/primer-blast/): *ACADS*, 5’-AGGGCCTGGCGGCAGTTACA-3‘ (forward), 5‘-CGCAGCCACGGCTGATCTCC-3‘ (reverse); *ACADM*, 5’- TGCCCTGGAAAGGAAAACTTTCGG-3‘ (forward), 5‘-ACCTCCCAAGCTGCTCTCTGGT-3‘ (reverse); *ACADL*, 5’- TGGCAAAACAGTTGCTCACCTACA-3‘ (forward), 5‘-GCAAGCAGTGGCGGAGTCCA -3‘ (reverse); *ACADVL*, 5’-GGGCTTCATGAAGGAACCTGGAG-3‘ (forward), 5‘-CTAGCAGGAGGCCAGCATTCC-3‘ (reverse); *GAPDH*, 5‘-GATCATCAGCAATGCCTCCTGC-3‘ (forward), 5‘-ACAGTCTTCTGGGTGGCAGTGA-3‘ (reverse); *YWHAZ*, 5‘-GCAACCAACACATCCTATCAGAC-3‘ (forward), 5‘-TTCTCCTGCTTCAGCTTCGTC-3‘ (reverse); *PPIA*, 5‘-GGATTTGGTTATAAGGGTTCC-3‘ (forward), 5‘-CAGTCTTGGCAGTGCAGAT-3‘ (reverse). The reference gene index (RGI) was calculated as arithmetic mean of *GAPDH*, *YWHAZ* and *PPIA* mRNA in each sample. Fold changes were calculated using the ΔΔCt method.

### Western blotting

Cell and tissue extracts were prepared in lysis buffer (50 mmol/L Tris-HCl (pH = 8), 150 mmol/L NaCl, 0.2% SDS, 1% NP-40, 0.5% Deoxycholat, 1 mmol/L PMSF and protease- and phosphatase inhibitors (Roche Diagnostics, Manheim, Germany). Protein samples (10 μg per lane) were separated on 12.5% PAGE–SDS gel, transferred (Biometra, Göttingen, Germany) on nitrocellulose membranes (Whatman, Dassl, Germany), exposed to primary antibodies overnight at 4°C (ACADS, ACADM, ACADL, ACADVL, Sigma Aldrich, Steinheim, Germany; GAPDH, Ambion, Invitrogen, Karlsruhe, Germany) followed by exposure to fluorochrome-conjugated secondary antibodies (Li-COR Bioscience, Bad Homburg, Germany). For quantification of ACADS, ACADM, ACADL, ACADVL and GAPDH protein levels the Odyssey infrared imaging system (Li-COR Bioscience, Bad Homburg, Germany) was used.

### *In situ* assay of FAO in LCL

The *in situ* assay of fatty acid beta-oxidation was carried out according to the method described by Ensenauer et al. [16]. Cell-type specific adaptations included counting of LCLs immediately before the assay using an automated cell counter (Countess, Invitrogen, OR, USA). After permeabilisation of 5 x 10^5^ cells/cell line (triplicates) with digitonin (10 μg/mL) (Sigma-Aldrich, Steinheim, Germany) for five min, LCLs were treated with 500 μL incubation buffer containing 100 μmol/L oleic acid (Sigma-Aldrich, Steinheim, Germany) for 160 min in a water bath at 37°C (GFL 1083, Hilab, Düsseldorf, Germany) while shaking gently. Acylcarnitines were extracted from both incubation medium and cell lysates by protein precipitation/desalting and subjected to solid-phase extraction. Acylcarnitines analysed by ESI-MS/MS were normalised to the corresponding citrate synthase activity.

### Citrate synthase (CS) activity measurement

5 x 10^5^ LCL cells were lysed in 200 μL 1% Triton X-100 (Sigma-Aldrich, Steinheim, Germany). CS activity was measured by adding 20 μL 5,5-dithiobis-(2-nitrobenzoic acid) (DTNB) (6 mmol/L dissolved in CS buffer) (Sigma-Aldrich, Steinheim, Germany), 20 μL acetyl-CoA (5 mmol/L dissolved in ddH2O) (Sigma-Aldrich, Steinheim, Germany), 520 μL CS buffer (1 mmol/L EDTA (Calbiochem, San Diego, CA, USA); 100 mmol/L KCl (Roth, Karlsruhe, Germany); 50 mmol/L Tris/HCl, pH = 8.0) (Applichem GmbH, Darmstadt, Germany) and 20 μL of the sample in a quartz cuvette. After 5 min incubation at RT, to avoid unspecific reactions, 20 μL oxaloacetic acid (Sigma-Aldrich, Steinheim, Germany) was added to start the reaction. The absorption was spectrophotometrically measured at 412 nm for 5 min every 30 sec to gather linear substrate formation (DU 800 Spectrophotometer, Beckmann Coulter, Krefeld, Germany).

### Loading of Huh7 cells with palmitic acid-BSA

Assay conditions for preparation of palmitic acid-BSA conjugates and loading of Huh7 cells with palmitic acid-BSA in Huh7 cells, using Seahorse technology, were adopted from the Seahorse user protocols (http://seahorsebio.com/resources/tech-writing/protocol-fatty-acid-oxidation.pdf, Seahorse Bioscience Inc., North Billerica, MA, USA). Palmitic acid (Sigma-Aldrich, Steinheim, Germany) was bound to fatty acid free BSA (Sigma-Aldrich, Steinheim, Germany) (molar ratio palmitic acid: BSA = 6:1) for solubilisation. Briefly, sodium palmitic acid was solubilised in 150 mmol/L sodium chloride at 70°C in a water bath; BSA was dissolved in 150 mmol/L sodium chloride at 37°C with continuous stirring. Solubilised palmitic acid was added to BSA at 37°C with continuous stirring. The pH was adjusted to 7.4 with 1 mol/L NaOH. Cells treated with dox were seeded in 6-well plates at a density of 250,000 cells / well and cultured in a 5% CO_2_ incubator at 37°C. After two days, growth medium (DMEM medium containing 10% FBS, 1% penicillin-streptomycin and 0 ng/mL, 5 ng/mL or 10 ng/mL doxycycline) was changed to assay medium (111 mmol/L NaCl, 4.7 mmol/L KCl, 2 mmol/L MgSO_4_, 1.2 mmol/L Na_2_HPO4, 0.5 mmol/L carnitine (all components from Sigma-Aldrich, Steinheim, Germany) at a final volume of 1.8 mL. After 60 min 0.2 mL 2 mmol/L palmitic acid-BSA was added. At baseline and after 7, 14, 21 and 28 min 20 μL supernatant was given on a 6 mm filter paper punch. Cells were washed with PBS and harvested by scraping in 300 μL ice-cold 100% methanol. Both, supernatant and cells were shock frozen in liquid nitrogen.

### Measurement of oxygen consumption

The Seahorse Extracellular Flux Analyzer XF96 (Seahorse Bioscience, North Billerica, MA, USA) measures oxygen consumption rate (OCR) in a 96 well format by sensing changes in oxygen content in a < 3 μL volume above the plated cells with a fluorescence biosensor. The measurements are non-invasive and made in short and repeated intervals. An assay medium composed of 111 mmol/L NaCl, 4.7 mmol/L KCl, 2 mmol/L MgSO_4_, 1.2 mmol/L Na_2_HPO_4_, 0.5 mmol/L carnitine (Sigma-Aldrich, Steinheim, Germany) and pH 7.4 was used in the XF analysis. The cells were seeded in a collagen pre-treated XF96 96 well cell culture microplate (Seahorse Bioscience, North Billerica, MA, USA) at 10,000 cells/well in 80 μL of growth medium (see cell culture methods) and incubated 2 days at 37°C in a humidified atmosphere of 5% CO_2_. Prior to assay, growth medium was removed and replaced by 140 μL of assay medium. The cells were preincubated under these conditions for 24h at 37°C in air. After four consecutive baseline OCR measurements palmitic acid was injected as a BSA-palmitic acid conjugate in a final concentration of 200 μM. To ensure that palmitic acid was metabolised in FAO and its products were used for respiration, the inhibitory effect of etomoxir on CPT-I was used which blocks the intake of palmitic acid into the mitochondrial matrix. Etomoxir (Sigma-Aldrich, Steinheim, Germany) was injected after a further 28 min at a final concentration of 50 μM.

### Extraction of acylcarnitines from Huh7 cells

Cells harvested in 300 μL 100% methanol were lysed in an ultrasonic bath (SONOREX SUPER RK 106 Bandelin, Berlin, Germany) for 10 secs, followed by shaking at 4°C for 20 min at 1500 rpm (Thermomixer comfort, Eppendorf, Hamburg, Germany). Cell debris was spun down (14.000 rpm, 10 min, 4°C, Eppendorf 5417 R, Hamburg, Germany) and supernatant was collected and vacuum dried in a speed vac (Savant SPD 111V SpeedVac Concentrator, Thermo Scientific, Dreieich, Germany) for 120 min. Dried pellets were resuspended in 100 μL 5 mmol/L NH_4_Ac in LC-MS methanol with internal standard (Chromsystems, Gräfelfing, Germany) containing amino acids (Alanine, Arginine, Aspartic acid, Citrulline, Glutamic acid, Glycine, Leucine, Methionine, Ornithine, Phenylalanine, Tyrosine, Valine) and acylcarnitines (C0-Carnitine, C2-Carnitine, C3-Carnitine, C4-Carnitine, C5-Carnitine, C5DC-Carnitine, C6-Carnitine, C8-Carnitine, C10-Carnitine, C12-Carnitine, C14-Carnitine, C16-Carnitine, C18-Carnitine), and filtered through a Millipore filter plate (Billerica, MA, USA) by centrifugation at 1,500 g for 20 min. Flow-through was collected in glass vials (Chromacol, Herts, UK) and stored at -80°C until measurement.

### Extraction of acylcarnitines from Huh7 cell culture supernatant

Filter paper punches soaked with 20 μL cell culture supernatant flow-through were vacuum dried in a speed vac (Savant SPD 111V SpeedVac Concentrator, Thermo Scientific, Dreieich, Germany) for approximately 45 min. 100 μL 5 mmol/L NH_4_Ac in LC-MS methanol containing internal standard (see above) was added to the dried filter paper punches which were shaken for 30 min at full speed and room temperature (Thermomixer comfort, Eppendorf, Hamburg, Germany). The supernatant was transferred into a Millipore filter plate and filtered by centrifugation at 1,500 g for 20 min. Flow-through was collected in glass vials and stored at -80°C until measurement.

### Acylcarnitine analysis in supernatant and Huh7 cell extracts

Chromatographic separations of metabolites in supernatants and cell extracts were conducted on a ZIC-HILIC column (150 x 4.6 mm, 5 μm) (Merck, Darmstadt, Germany). A QTRAP 5500 (AB Sciex, Framingham, MA, USA) triple-quadrupole tandem mass spectrometer with Turbo V spray electron spray interface was used in positive ion mode for detection. Analyst and MultiQuant software (AB Sciex, Framingham, MA, USA) were used for data acquisition and data processing. Limit of quantification was defined as signal/noise ratio > 9. Acylcarnitine measurements were normalised to the protein amount per well, data of all single measurements are given in S1A–S1D Table.

### Statistical analysis

All data are expressed as mean ± SD if not stated otherwise. *ACADS* mRNA expression of LCLs and Huh7 cells were normalised to the reference gene index. Fold changes were calculated using the ΔΔCt method. Differences of gene expression in LCLs and Huh7 cells were assessed by two-tailed one sample t-test. C4-acylcarnitine levels in LCLs from the metabolite-flux assay were compared using Mann-Whitney-U test. Kruskal-Wallis one-way ANOVA with Dunn’s post hoc test was used to compare C4-acylcarnitine accumulation in shACADS and shNTC Huh7 cells. Data were analysed by two-way ANOVA with Bonferroni post hoc test for multiple comparisons against control to study the effects of different knockdowns over time. To quantify differences in the dynamics of time-dependent metabolite flux FAO was modelled in a linear cascade of subsequent, irreversible first-order reactions using ordinary differential equations with mass action kinetics [19–21] using acylcarnitine concentrations as a reflection for acyl-CoA concentrations. Model simulations were compared to acylcarnitine data on log_10_-scale to account for log-normally distributed metabolite concentrations and reaction kinetics [22], resulting in model parameters optimally describing the measured data [23]. A detailed description of equations and model selection is presented in S1 Text. All statistical and quantitative dynamical modelling analyses were performed using GraphPad Prism5 (GraphPad software, La Jolla, CA, USA), MATLAB (R2012a, The Mathworks Inc., Natick, MA) and the Data2Dynamics software package [24].

## Results

### Reduced ACADS expression levels and altered FAO rate in LCLs from subjects homozygous for the rs2014355 and the coding rs1799958 minor allele

First, we tested the hypothesis that common *ACADS* variants modulate ACADS enzyme function in FAO [10–14]. We measured acylcarnitine flux in LCLs genotyped for two lead SNPs identified by mQTL; the noncoding variant rs2014355T>C and the non-synonymous coding variant rs1799958G>A (c.625G>A, p.G209S) which are in perfect linkage disequilibrium (LD = 1.0, 1000 Genomes Phase 3 [25]; confirmed by sequencing in our LCLs). Absence of further *ACADS* non-synonymous mutations was confirmed by sequencing. We included LCLs homozygous for the major or minor allele of rs2014355T>C and rs1799958G>A (c.625G>A, p.G209S), respectively.

**Fig 1:**
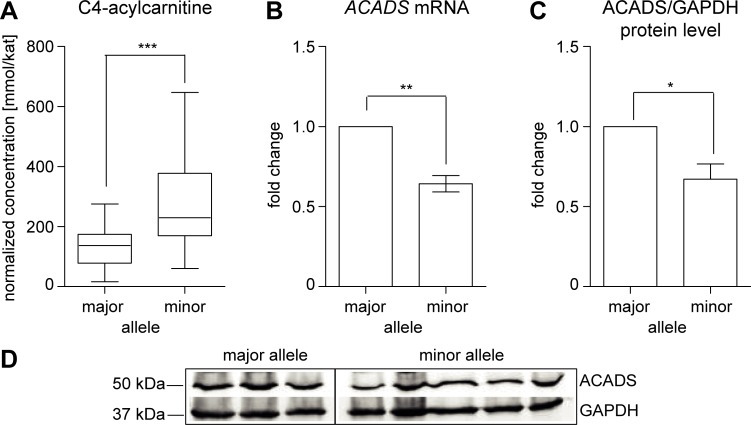
Phenotype of LCLs from donors of the KORA population cohort. Analysis was performed on samples from three homozygous major T- and G-allele and five homozygous minor C- and A-allele carriers of the rs2014355T>C and rs1799958G>A (c.625G>A, p.G209S) variants. (A) Genotype-dependent C4-acylcarnitine levels in LCLs after 160 min incubation with 100 μmol/L oleic acid. Acylcarnitine levels were normalised to the citrate synthase activity. Values of 3–6 independent experiments (each in triplicates) for each of the eight analysed LCL are expressed as box plots (Boxes extend from first quartile to third quartile; median for each genotype is indicated as a horizontal line; whiskers are drawn equal to 1.5 times the interquartile distance). *** = p < 0.001; Mann-Whitney-U test. (B) mRNA samples were isolated in four independent experiments from each of the eight cell lines and analysed by RT-qPCR. The reference gene index (RGI) was calculated as arithmetic mean of *GAPDH* and *YWHAZ* in each LCL sample. Fold changes were calculated using the ΔΔCt method. (C) Protein samples were isolated in four independent experiments from each of the eight individual cell lines and western blot analysis of ACADS protein levels was carried out in 10 μg protein lysates of LCLs. Band intensities were analysed and quantified using the Odyssey IR Imaging System (LI-COR, Bioscience) and ACADS was normalised to GAPDH. Values are mean ± SD. * = p < 0.05; ** = p < 0.01; two-tailed, one-sample t-test. (D) Exemplarily western blot of one independent experiment analysing ACADS expression in the three major and five minor allele carriers.

An *in situ* assay of FAO with oleic acid loading revealed a significant 1.8-fold increase of total C4-acylcarnitine levels in LCLs homozygous for the minor allele (Fig 1A). Next, we assessed *ACADS* mRNA and ACADS protein expression levels that may explain the reduced C4-acylcarnitine conversion found in LCLs from minor allele carriers. Both, mRNA (Fig 1B) and protein levels (Fig 1C and 1D) were significantly reduced in LCLs homozygous for the minor allele as compared to controls homozygous for the major allele.

### *In vitro* model of reduced ACADS expression in Huh7 hepatocytes reflects the genotype-dependent metabolic C4-acylcarnitine phenotype

**Fig 2:**
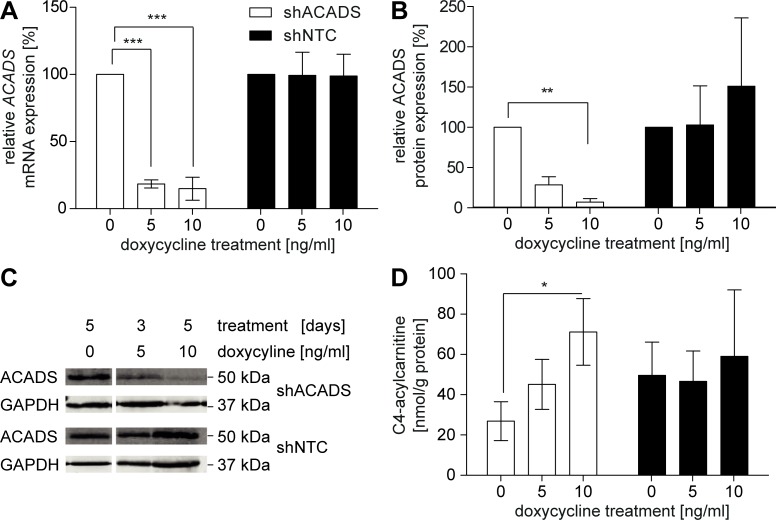
Phenotypes of the shACADS and shNTC knockdown Huh7 cells. Doxycycline-induced knockdown of ACADS resulted in a long-term decrease of protein abundance and mRNA expression, and an intracellular accumulation of C4-acylcarnitine, which reflects the ACADS substrate C4-CoA. (A) RT-qPCR analysed mRNA of four independent experiments, shown as mean ± SD. (B, C) Western blot analysis of ACADS protein in shACADS and shNTC knockdown Huh7 cell lysates. Western blotting analysed 10 μg of cell lysate per sample depicted exemplary for one (C) of four experiments (B). ** = p < 0.01 (one sample t-test). mRNA (A) and Protein (B) were harvested after 3 or 5 days of treatment with 0, 5, and 10 ng/mL doxycycline, respectively. *** = p < 0.001 (one-sample t-test). (D) Intracellular C4-acylcarnitine measurement in stably transduced Huh7 cells. Values of four independent experiments are expressed as mean ± SD. * = p< 0.05 (Kruskal-Wallis one-way ANOVA with Dunn’s post hoc test). NTC = non-target control.

Next, we assessed how gradual changes of ACADS expression levels modulate FAO in a cell type with high FAO-capacity–here Huh7 hepatocytes–which may contribute to the observed association of plasma C4-acylcarnitine levels with the common *ACADS* genotypes FAO [10–14]. We established an inducible ACADS knockdown in Huh7 cells as *in vitro* model enabling the investigation of gradually reduced ACADS expression levels on intra- and extracellular acylcarnitine levels. We first confirmed the validity of the Huh7 cell system as a model for FAO by assessing the oxygen consumption rate in Huh7 cells after palmitic acid loading (S1A and S1B Fig). Huh7 cells were stably infected using a lentiviral expression system generating a doxycycline-inducible shRNA ACADS knockdown (shACADS). Evaluating the efficiency of the doxycycline-inducible *ACADS* knockdown by RT-qPCR we found a significant reduction of *ACADS* mRNA levels in the intermediate (shACADS^med^, doxycycline 5 ng/mL) and maximal (shACADS^max^, doxycycline 10 ng/mL) shACADS Huh7 cells (82% and 84%, respectively, p < 0.001, Fig 2A) as compared to the null knockdown (shACADS^null^, doxycycline 0 ng/mL). In western blot analyses, we found gradual 70% and 93% reduction of ACADS protein levels for shACADS^med^ and shACADS^max^ Huh7 cells, respectively (Fig 2B and 2C). Doxycycline-treatment of Huh7 cells stably transduced with a non-targeting control shRNA (shNTC) revealed no effect on both, protein and mRNA levels (Fig 2A–2C). We found no differences when comparing mRNA expression levels in shACADS versus shNTC cells without doxycycline treatment (p = 0.96, one-sample t-test, n = 4), proving that the lentiviral shRNA construct is solely active upon doxycycline treatment without any leakiness. Moreover, we found that the knockdown does not affect mRNA (S1C Fig) and protein (S1D and S1E Fig) expression levels of medium- and long-chain acyl-CoA dehydrogenase *ACADM*, *ACADL*, and *ACADVL*.

Analysis of baseline intracellular acylcarnitine levels in the shACADS Huh7 cell model revealed a knockdown-dependent accumulation of C4-acylcarnitine (Fig 2D). Maximal knockdown in the shACADS cells caused a significant 2.6-fold increase of baseline C4-acylcarnitine levels (p < 0.05) as compared to shACADS^null^. The shACADS Huh7 hepatocyte knockdown model in this respect reflects the rs2014355/rs1799958 locus associated changes in C4-acylcarnitine levels also presented in the LCL model. No significant differences were observed in shNTC cells upon doxycycline-treatment, proving ACADS-specificity of the observed effect (Fig 2D). We found no ACADS-dependent significant differences in baseline levels of any other measured acylcarnitine in either cell model (time-point t = 0, S2 and S3 Figs). Moreover, reflecting the results from numerous GWAS FAO [10–14] we found a significant 2.1-fold decrease of the intracellular C3/C4-acylcarnitine ratio (p < 0.05, S1F Fig) in shACADS^max^ cells as compared to shACADS^null^ cells.

### Model-based quantification of time-dependent intracellular acylcarnitine levels in shACADS cells after palmitic acid loading infers novel FAO signatures

**Fig 3:**
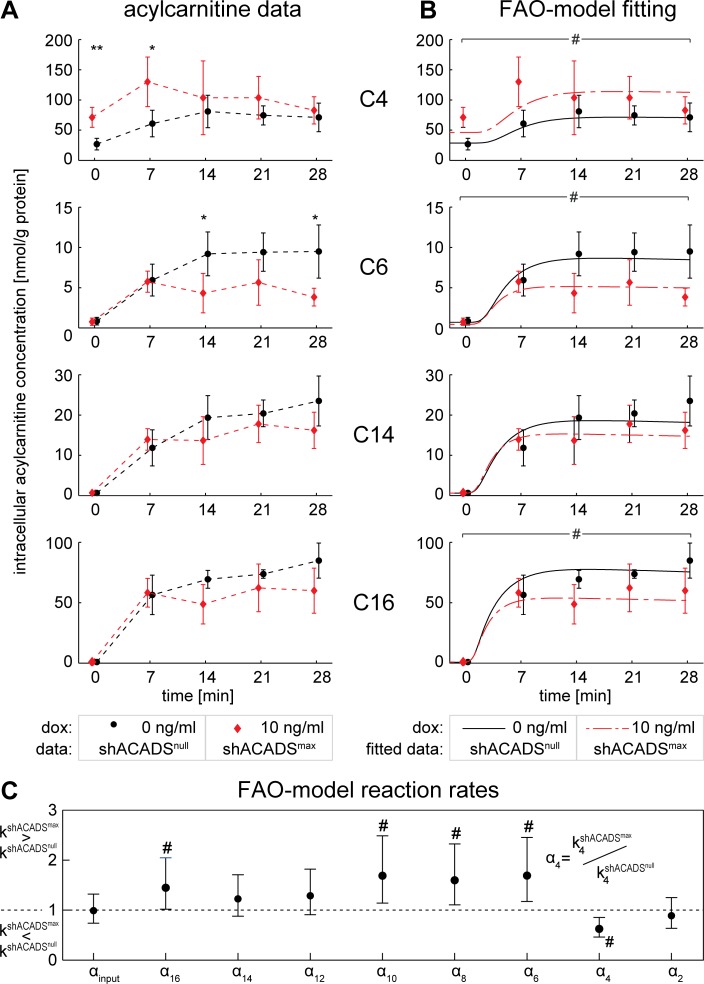
Time-course of intracellular acylcarnitine levels in ACADS knockdown cells—comparison of distinct time-points and kinetic differences. (A) Intracellular acylcarnitine levels, representing acyl-CoAs with corresponding chain length, were extracted and measured before palmitic acid loading and 7, 14, 21 and 28 min after loading in shACADS knockdown Huh7 cells, i.e. shACADS^null^ and shACADS^max^ cells treated with 0 and 10 ng/ml doxycycline (dox), respectively, for shRNA induction. Shown are levels of C4-acylcarnitine as primary outcome, C14-, C6-acylcarnitines representing long- and medium-chain acylcarnitines, and C16-acylcarnitine (note that data for acylcarnitines C2 to C16, including the here presented, are given in S2 Fig). Values of four independent experiments are shown as mean ± SD (original data of single measurements are given in S1A Table). Time point specific comparison between shACADS^null^ and shACADS^max^, were performed using t-tests; *p < 0.05 and **p < 0.01. Results for all acylcarnitines in S2 and S3 Figs for cells treated with shACADS and shNTC, respectively. (B) Results from fitting of linear FAO model to the measured data (note that data for acylcarnitines C2 to C16 are given in S2 Fig). (C) FAO model based quantification of differences in acylcarnitine flux. shACADS^null^ and shACADS^max^ are compared by the ratio α = k^shACADSmax^ / k^shACADSnull^, reaction rates k are derived from the FAO model fits. α-values from best model fits are represented as dots with respective 95% confidence intervals. (B+C) # indicates significant difference (p < 0.05) between reaction rate k^shACADSmax^ and k^shACADSnull^ of the respective knockdown experiments. For details of dynamical modelling, see materials and methods, S4–S6 Figs and S1 Text.

To assess the effect of reduced ACADS enzyme expression levels on FAO kinetics, we leveraged the shACADS Huh7 cell model and measured the changes of intra-cellular acylcarnitine levels over time after incubation of cells with palmitic acid as FAO pathway substrate. We focused analysis on even-numbered intermediates of the FAO cascade, as palmitic acid is an even-numbered, saturated fatty acid. Intracellular acylcarnitine concentrations increased after exposure of cells to palmitic acid, suggesting increased FAO (Fig 3A, S2 and S3 Figs, S1 Table). In shACADS^max^ cells, C4-acylcarnitine levels are further increased at all time points as compared to shACADS^null^ knockdown, significant effects are observed at baseline and seven min after palmitic acid induction (p < 0.01 and p < 0.05, respectively, Fig 3A). Notably, the measured levels of longer chain acylcarnitines were lower in shACADS^max^ knockdown at most time points as compared to shACADS^null^, with significant effects found for some C6-, C8-, C10- and C12-acylcarnitines time points (Fig 3A, S2 Fig). Additionally, comparing time courses of metabolite levels in shACADS^null^, shACADS^med^ and shACADS^max^ cells confirms the gradual changes in ACADS expression; i.e. intermediate metabolite levels in the intermediate knockdown cells shACADS^med^ particularly for C10-, C8-, and C6-acylcarnitines (S2 Fig). Any influence of doxycycline-treatment was excluded by non-target shRNA experiments (S3 Fig).

Next, we aimed to approximate ACADS knockdown-dependent changes of FAO-kinetics from the here measured intracellular acylcarnitines C16 to C2 profiles (as FAO intermediate metabolite proxies [19–21]). Therefore, we first fitted a simplified mathematical FAO model (S1 Text) to the experimental data (Fig 3B, S4 and S5 Figs). We used this mathematical FAO model to calculate reaction rates for different steps of FAO and found that shACADS^max^ results in a significant decrease (p < 0.05, Fig 3C) of the reaction rate for C4 to C2 conversion, reflecting the predominant role of ACADS for this reaction [26] and the observed increase of C4-levels. Supporting the sensitivity and specificity of the regulated shACADS cell model, also intermediate shACADS^med^ knockdown was sufficient to decrease specifically the C4 to C2 reaction rate (p < 0.05, S6 Fig). Interestingly, we also found a significant increase of the reaction rates for C16, C10, C8 and C6-acylcarnitine conversion (p < 0.05, Fig 3C) upon maximal ACADS knockdown, reflecting the observed decreased acylcarnitine levels in shACADS^max^ cells (S2 Fig). ACADS knockdown did not affect C2-acylcarnitine levels (S2, S3, S5 and S6 Figs).

### Extracellular shACADS cell acylcarnitine profiles suggest direct contribution to plasma mQTL phenotype

**Fig 4:**
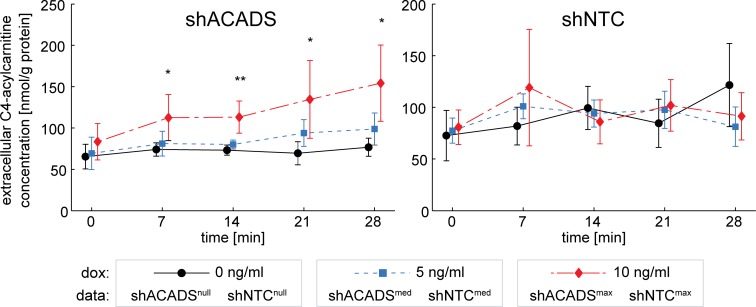
Time courses of extracellular C4-acylcarnitine after palmitic acid loading. Palmitic acid was added to induce FAO in shACADS and shNTC Huh7 cells. C4-acylcarnitine, which accumulates within the cell due to the ACADS knockdown, also accumulates in the supernatant. Gradual expression of shRNA was induced using 0, 5 and 10 ng/mL doxycycline. C4 was measured in supernatants before palmitic acid loading and after 7, 14, 21 and 28 min. Values of four independent experiments are expressed as mean ± SD. (original data of single measurements are given in S1C and S1D Table). **: p < 0.01, *: p < 0.05 comparison between shACADS dox 0 ng/mL and shACADS dox 10 ng/mL (two-sample t-test).

Finally, we determined extracellular C4-acylcarnitine levels in the cell culture medium supernatant (Fig 4). Extracellular baseline C4-acylcarnitine levels were higher than intracellular levels (65.4±17.2 and 26.9±9.7 nmol/g protein, Figs 4 and 3A, respectively, S1 Table), which may be attributed to an efflux of intracellular C4 before ACADS knockdown and palmitic acid loading. However, matrix effects cannot be excluded, challenging direct comparability of absolute levels measured in cell extracts *vs*. medium.

At baseline no significant ACADS-dependent differences of extracellular C4-acylcarnitine levels were found in shACADS^null^, shACADS^max^ and shNTC cells (time-point t = 0, Fig 4), whereas basal intracellular C4-acylcarnitine levels were increased 2.65-fold (p < 0.01, time-point t = 0) in shACADS^max^ as compared to shACADS^null^ cells (Figs 2D and 3A). In both shACADS^null^ and shNTC cells no effect of palmitic acid loading on extracellular C4-acylcarnitine levels was observed (Fig 4), despite the observed intracellular increase (Fig 3A). Strikingly, in shACADS^max^ cells we found a significant increase of extracellular C4-acylcarnitine levels as compared to shACADS^null^ cells at each of the four measured time points after palmitic acid loading (p < 0.05 or p < 0.01; Fig 4). This ACADS-dependent, significant increase of extracellular C4-acylcarnitine (Fig 4) reflects the significant increase of intracellular C4-acylcarnitine levels (Fig 3A).

## Discussion

Defining the mechanistic basis of how common gene variants affect QTLs or disease states can be difficult and demanding. Moreover, GWAS-inferred variants are frequently noncoding and suggested to affect the expression level of nearby or other genes. Population-based mQTL analysis identified altered plasma levels of C4-acylcarnitine levels associated with a common *ACADS* locus [10–14] in high linkage with previously described mutations [27,28]. *ACADS* codes for an enzyme involved in mitochondrial FAO, an important energy metabolism pathway, and these *ACADS* locus variants may affect the rate of FAO. Here, we selected the *ACADS* locus for assessing this association at the cellular and molecular level using two cell models, donor-specific genotyped LCLs and an inducible lentiviral hepatocyte knockout cell line. Combining these two cell models allowed the generation of a data set enabling an *in vitro* assessment on how a gradual, genotype-specific modulation of gene-expression contributes to the FAO pathway and phenotypes.

Decreased plasma C3/C4-acylcarnitine ratios in subjects carrying the common rs2014355 variant minor allele at the *ACADS* locus have been reported (minor allele frequency = 26%; 1,000 Genome Phase 1; EUR [25]) [10,11]. This suggested that there may be an effect on FAO capacity via the ACADS enzyme which catalyses the conversion of C4-CoA into C2-CoA [15]. We analysed intracellular acylcarnitine levels after oleic acid loading of LCLs selected for the perfectly linked noncoding rs2014355 and non-synonymous coding rs1799958 (c.625G>A, p.G209S) variants. The specific accumulation of C4-acylcarnitine—as proxy-metabolite of the ACADS substrate C4-CoA [19,20,29,30]—in LCL homozygous for the minor allele, supports that GWAS [10,11] and candidate gene study [31] association of the *ACADS* locus with plasma C4-acylcarnitine level may be caused by reduced intracellular conversion of C4-CoA species into acetyl-derivatives [10–14]. Corroborating this hypothesis, we observed reduced ACADS protein levels in LCL with the minor allele, possibly due to miss-folding or impaired thermo-stability of ACADS protein previously observed *in vitro* for the rs1799958 (c.625G>A, p.G209S) minor allele [27,32–34]. Moreover, we observed reduced *ACADS* mRNA expression levels in LCL homozygous for the minor allele. This suggests that variants at the *ACADS* locus may interfere with *cis*-regulatory mechanisms of ACADS expression.

Our data highlight that, going beyond eQTL analysis—frequently and successfully performed in LCL [35–39] -, also genotype-dependent differences in metabolic phenotypes related to a differentially expressed gene, such as FAO and ACADS, can be experimentally studied in the LCL cell model. Yet, the reported genotype associations with plasma acylcarnitine levels [10,11] may not result from lymphocytes, from which LCL are derived and which oxidise fatty acids although the main energy substrates are glucose and glutamine [40]. Rather, also tissues where FAO serves as substantive energy source, such as muscle, adipose tissue or particularly liver, where genotype dependent ACADS expression was found [41], may be a source of plasma acylcarnitine levels.

Here, we used gradual knockdown of endogenous ACADS in the human Huh7 hepatocyte cell line as cell model to further assess our findings in LCL. We observed that gradual reduction of ACADS expression levels leads to an accumulation of intracellular C4-acylcarnitine species. Moreover, we found a concurrent increase of C4-acylcarnitine levels in the medium along with the intracellular accumulation of C4-acylcarnitine. This increase in extracellular space was highly specific for C4-acylcarnitine and not observed for any other acylcarnitine and relates to the observation in human plasma with elevated C4-acylcarnitine levels associated with the rs2014355 genotype in GWAS [10–12] and the LCL phenotype. Notably, in shACADS knockdown without palmitic acid loading we found increased intracellular, but not increased extracellular C4-acylcarnitine levels, suggesting that acylcarnitine release from cells may be regulated by fatty acid stimulation or fatty acid availability in the cells and supporting the importance to consider time-course experiments. It has been proposed that the organic cation transporter 2 (*OCTN2*) may mediate the efflux of acylcarnitines from cells, but a recent study failed to confirm this [42]. Thus, the identity of plasma membrane transporters involved in the export of these metabolites remains to be determined. For such studies, the here described Huh7 cell model may be useful. Moreover, such a lentiviral approach is suitable for the assessment of metabolic alterations caused by genotype-specific alterations in mRNA levels in an appropriate biological context. The combination of such cells models can complement recent advancements in genome editing [43,44] and provides a practical, efficient analysis of genotype-phenotype relationships and underlying mechanisms.

How reduced ACADS expression modulates the overall kinetics of FAO is largely unknown. Further leveraging the hepatocyte shACADS model, we assessed time- and ACADS-dependent changes in acylcarnitine levels after exposure of the cells to palmitic acid as a substrate. We applied a mathematical FAO model tailored to our experimental data, as differences of measured metabolite and model complexity limits transfer of models described literature [21,45,46], and trimmed the initial general description of the linear FAO pathway down to the reactions essential to describe the observed data. Such an approach enables data analysis on pathway dynamics over time taking into account the reaction kinetics between fatty acids; a type of information that cannot be obtained from statistics on single metabolite alone. Our model is of course a simplified representation of the FAO pathway revealing knockdown-specific differences in metabolite dynamics, but not delivering kinetic rates. However, using a similar strategy we previously modelled plasma acylcarnitine levels in a human study with a starvation-induced increase in FAO resulting in reasonable approximations [47]. Comparing the dynamic changes resulting from Huh7 liver cell ACADS knockdown we obtained a significant decrease in the C4-related reaction rates, reflecting both, the rs2014355 genotype-specific ACADS gene expression and C4 levels in LCL and GWAS data [10,11,31]. We note that in Huh7 experiments we assessed palmitic acid C16:0, but in LCL oleic acid C18:1 requiring an auxiliary upstream oxidation step, yet after the first cycles of FAO oleic acid yields the same intermediates as palmitic acid. Further experiments are necessary for direct comparison on how different fatty acids may affect the observed effects. We did not observe a decrease of C2 levels as a product of the ACADS reaction. Acetyl-CoA or its carnitine derivative can also originate from a number of other pathways and thus may not be a good indicator of an altered FAO flux. The unexpected increase in medium- and long-chain carnitine reaction rates and decreased specimen levels may reflect the C4 accumulation with effects on upstream products or reactions. Changes in the protein levels of medium- and long-chain acyl-CoA dehydrogenases *ACADM*, *ACADL* and *ACADVL* causing these effects were excluded by Western-blot analysis leaving changes in enzyme activity as the likely cause. Further experiments are needed to assess such effects in other cell types than liver Huh7, such as the here analysed LCL or in other cell types with importance for FAO such as adipose tissue.

In summary, we have shown genotype-dependent changes of C4-acylcarnitine levels in LCLs homozygous for the minor allele of a common *ACADS* locus. Impaired FAO may be explained by both, reduced mRNA and protein expression levels which may be affected by different mechanisms of noncoding variants tagged by rs2014355 and the rs1799958A (p.209S) coding variant, respectively. Using the Huh7 hepatocyte cell line we observed that an impaired palmitic acid-induced FAO flux—induced by a specific, gradual reduction of ACADS expression levels–results in an intracellular accumulation of C4-, medium- and long-chain acylcarnitine species, and an elevated efflux of C4-acylcarnitines. Regarding the limited availability of relevant cell systems, i.e. genotyped primary human cells [5] essential for functional analysis, the cell models presented here may prove helpful to assess the functional consequences of common genetic variants.

## Supporting information

S1 Fig Characterisation of the Huh7 cell model. (A-B) Increase of oxygen consumption after palmitic acid and inhibition by the FAO inhibitor etomoxir shows the validity of the Huh7 cell model to assess FAO. Change in oxidative consumption rate OCR (pmol/min) after stimulation and inhibition of FAO in Huh7 cells. (A) Extracellular O_2_ measurement in Huh7 cells after 24h starvation in assay medium, injection of palmitic acid (200 μM) [P] and injection of CPT-1 inhibitor etomoxir (50 μM) [E]. Values of eight parallel measurements are expressed as mean ± SD. (B) Maximum increase of baseline OCR after palmitic acid injection and maximal decrease of OCR after etomoxir injection. Values of eight parallel measurements are expressed as mean + SD. ** = p < 0.01; * = p < 0.05; (Repeated measures ANOVA (Friedman test)). OCR = oxidative consumption rate. (C-E) Doxycycline-induced knockdown of ACADS does not affect mRNA expression levels or protein abundance of medium and long chain acyl-CoA dehydrogenases *ACADM*, *ACADL* and *ACADVL* in shACADS knockdown Huh7. (C) RT-qPCR analysed *ACADM*, *ACADL* and *ACADVL* mRNA of four independent experiments, shown as mean ± SD. (D-E) Western blot analysis of ACADM, ACADL and ACADVL proteins in shACADS knockdown Huh7 cell lysates. Western blotting analysed 10 μg of cell lysate per sample depicted exemplary for one (E) of four experiments (D). mRNA (C) and Protein (D-E) were harvested after 3 or 5 days of treatment with 0, 5, and 10 ng/mL doxycycline, respectively. One-sample t-test for (C) and (D) revealed no significant effect. (F) Decrease of intracellular C3/C4-acylcarnitine ratio in shACADS^max^ Huh7 cells. Intracellular C3- and C4-acylcarnitine measurement in doxycycline-induced Huh7 shACADS^null^ and shACADS^max^ cells (treated with 0 and 10 ng/mL doxycycline, respectively). Values of four independent experiments are expressed as box plots (Boxes extend from first quartile to third quartile; median is indicated as a horizontal line; whiskers are drawn equal to 1.5 times the interquartile distance). * = p < 0.05; two-tailed unpaired t-test.
(PDF)

S2 Fig Time courses of intracellular acylcarnitines after palmitic acid loading in shACADS Huh7 cells. Palmitic acid was added to induce fatty acid oxidation in shACADS knockdown cells. shACADS^null^, shACADS^med^ and shACADS^max^ cells treated with 0, 5 and 10 ng/mL doxycycline (dox), respectively, for shRNA induction. Intracellular acylcarnitines, assumed to represent acyl-CoAs with corresponding chain length, were extracted and measured before palmitic acid loading and after 7, 14, 21 and 28 min. Values of four independent experiments are expressed as mean ± SD (original data of single measurements are given in S1A Table). ND: concentration not measured. Time point specific comparison between shACADS^null^ and shACADS^max^ using t-test with **p < 0.01, *p < 0.05.
(PDF)

S3 Fig Time courses of intracellular acylcarnitines after palmitic acid loading in Huh7 cells with non-target shRNA. Palmitic acid was added to induce fatty acid oxidation in cells transduced with a non-target shRNA. shNTC^null^, shNTC^med^ and shNTC^max^ cells treated with 0, 5 and 10 ng/mL doxycycline (dox), respectively, for shRNA induction. Intracellular acylcarnitines, assumed to represent acyl-CoAs with corresponding chain length, were extracted and measured before palmitic acid loading and after 7, 14, 21 and 28 min. Values of four independent experiments are expressed as mean ± SD (original data of single measurements are given in S1B Table). ND: concentration not measured. Time point specific comparison between shNTC^null^ and shNTC^max^ using t-test with *p < 0.05.
(PDF)

S4 Fig Illustration of the mathematical fatty acid oxidation chain model. In each FAO reaction step of palmitic acid loaded Huh7 cells the carbon chain is shortened and C2 is produced. Fundamental chain and influx reactions for C16, C14-, C8- and C4-acylcarnitine are described by reaction rates (*k*_*16*_,*…*,*k*_*2*_,*k*_*input*_,*k*_*14in*_,*k*_*8in*_,*k*_*4in*_). For a detailed description of the model and the data-driven selection of influx reactions see methods section and S1 Text.
(PDF)

S5 Fig Model-based analysis of intracellular acylcarnitine time course data in cells with maximal ACADS knockdown. Results from fitting the linear fatty acid oxidation model to the null (shACADS^null^) and maximal (shACADS^max^) ACADS knockdown data. Intracellular acylcarnitine levels, representing acyl-CoAs with corresponding chain length, were extracted and measured before palmitic acid loading and 7, 14, 21 and 28 min after loading in shACADS knockdown Huh7 cells, i.e. shACADS^null^ and shACADS^max^ cells treated with 0 and 10 ng/ml doxycycline (dox), respectively, for shRNA induction. Values of four independent experiments are shown as mean ± SD (original data of single measurements are given in S1A Table). # indicates significant difference (p < 0.05) between reaction rate k^shACADSmax^ and k^shACADSnull^ of the respective knockdown experiments (see also Fig 3C).
(PDF)

S6 Fig Model-based analysis of intracellular acylcarnitine time course data in cells with intermediate ACADS knockdown. (A) Results from fitting the linear fatty acid oxidation model to the null (shACADS^null^) and intermediate (shACADS^med^) ACADS knockdown data. Intracellular acylcarnitine levels, representing acyl-CoAs with corresponding chain length, were extracted and measured before palmitic acid loading and 7, 14, 21 and 28 min after loading in shACADS knockdown Huh7 cells, i.e. shACADS^null^ and shACADS^med^ cells treated with 0 and 5 ng/ml doxycycline (dox), respectively, for shRNA induction. Values of four independent experiments are shown as mean ± SD (original data of single measurements are given in S1A Table). (B) FAO model-based quantification of differences in acylcarnitine flux dynamics. shACADS^med^ and shACADS^null^ are compared by the ratio α = k^shACADSmed^ / k^shACADSnull^, reaction rates k are derived from the FAO model fits. α-values from best model fits are represented as dots with respective 95% confidence intervals. (A+B) # indicates significant difference (p < 0.05) between reaction rate k^shACADSmed^ and k^shACADSnull^ of the respective knockdown experiments. For details of dynamical FAO modelling, see materials and methods, S4–S6 Figs and S1 Text. Compared to the null knockdown, in the intermediate knockdown reaction rate k_4_ is significantly decreased, whereas reaction rate k_8_ is significantly increased.
(PDF)

S1 Text Description of the fatty acid oxidation model. (PDF)

S1 Table **S1A Table Intracellular acylcarnitine levels in Huh7 with shACADS knockdown—original data**. Acylcarnitines were extracted as described in material and methods from the Huh7 cell line shACADS (shACADSnull, med and max indicates no treatment or treatment with 5 and 10 ng/mL doxycycline, respectively) before (time point 0 min) and after loading of the cells with palmitic acid (time points 7–28 min). In brief, after chromatographic separation of metabolites in the cell extracts triple-quadrupole tandem mass spectrometer was used for detection of metabolites, analyst and MultiQuant software for data acquisition and data processing. Limit of quantification was defined as signal/noise ratio > 9. The measured acylcarnitine levels are presented in nmol per g protein for each experiment. **S1B Table. Intracellular acylcarnitine levels in Huh7 cells with shNTC control knockdown—original data**. Acylcarnitines were extracted as described in material and methods from the Huh7 cell line shNTC (shNTCnull, med and max indicates no treatment or treatment with 5 and 10 ng/mL doxycycline, respectively) before (time point 0 min) and after loading of the cells with palmitic acid (time points 7–28 min). In brief, after chromatographic separation of metabolites in the cell extracts triple-quadrupole tandem mass spectrometer was used for detection of metabolites, analyst and MultiQuant software for data acquisition and data processing. Limit of quantification was defined as signal/noise ratio > 9. The measured acylcarnitine levels are presented in nmol per g protein for each experiment. **S1C Table. Extracellular C4-acylcarnitine levels in Huh7 with shACADS knockdown—original data**. C4-Acylcarnitines in the cell culture supernatant from the Huh7 cell line shACADS (shACADSnull, med and max indicates no treatment or treatment with 5 and 10 ng/mL doxycycline, respectively) before (time point 0 min) and after loading of the cells with palmitic acid (time points 7–28 min). In brief, after chromatographic separation of metabolites in the cell culture supernatant triple-quadrupole tandem mass spectrometer was used for detection of metabolites, analyst and MultiQuant software for data acquisition and data processing. Limit of quantification was defined as signal/noise ratio > 9. The measured acylcarnitine levels are presented in nmol per g protein for each experiment. **S1D Table. Extracellular C4-acylcarnitine levels in Huh7 cells with shNTC control knockdown—original data**. C4-Acylcarnitines in the cell culture supernatant from the Huh7 cell line shNTC (shNTCnull, med and max indicates no treatment or treatment with 5 and 10 ng/mL doxycycline, respectively) before (time point 0 min) and after loading of the cells with palmitic acid (time points 7–28 min). In brief, after chromatographic separation of metabolites in the cell culture supernatant triple-quadrupole tandem mass spectrometer was used for detection of metabolites, analyst and MultiQuant software for data acquisition and data processing. Limit of quantification was defined as signal/noise ratio > 9. The measured acylcarnitine levels are presented in nmol per g protein for each experiment.
(XLS)
