# Supplementary material for: Dynamic modelling of an ACADS genotype in fatty acid oxidation – Application of cellular models for the analysis of common genetic variants
Source: PLoS One. 2019 May 23;14(5):e0216110. doi: 10.1371/journal.pone.0216110 (PMC6532850; doi:10.1371/journal.pone.0216110)
Supplement: S1 Fig — (A-B) Increase of oxygen consumption after palmitic acid and inhibition by the FAO inhibitor etomoxir shows the validity of the Huh7 cell model to assess FAO. Change in oxidative consumption rate OCR (pmol/min) after stimulation and inhibition of FAO in Huh7 cells. (A) Extracellular O2 measurement in Huh7 cells after 24h starvation in assay medium, injection of palmitic acid (200 μM) [P] and injection of CPT-1 inhibitor etomoxir (50 μM) [E]. Values of eight parallel measurements are expressed as mean ± SD. (B) Maximum increase of baseline OCR after palmitic acid injection and maximal decrease of OCR after etomoxir injection. Values of eight parallel measurements are expressed as mean + SD. ** = p < 0.01; * = p < 0.05; (Repeated measures ANOVA (Friedman test)). OCR = oxidative consumption rate. (C-E) Doxycycline-induced knockdown of ACADS does not affect mRNA expression levels or protein abundance of medium and long chain acyl-CoA dehydrogenases ACADM, ACADL and ACADVL in shACADS knockdown Huh7. (C) RT-qPCR analysed ACADM, ACADL and ACADVL mRNA of four independent experiments, shown as mean ± SD. (D-E) Western blot analysis of ACADM, ACADL and ACADVL proteins in shACADS knockdown Huh7 cell lysates. Western blotting analysed 10 μg of cell lysate per sample depicted exemplary for one (E) of four experiments (D). mRNA (C) and Protein (D-E) were harvested after 3 or 5 days of treatment with 0, 5, and 10 ng/mL doxycycline, respectively. One-sample t-test for (C) and (D) revealed no significant effect. (F) Decrease of intracellular C3/C4-acylcarnitine ratio in shACADSmax Huh7 cells. Intracellular C3- and C4-acylcarnitine measurement in doxycycline-induced Huh7 shACADSnull and shACADSmax cells (treated with 0 and 10 ng/mL doxycycline, respectively). Values of four independent experiments are expressed as box plots (Boxes extend from first quartile to third quartile; median is indicated as a horizontal line; whiskers are drawn equal to 1.5 times the interquartile distance). [file pone.0216110.s001.pdf]

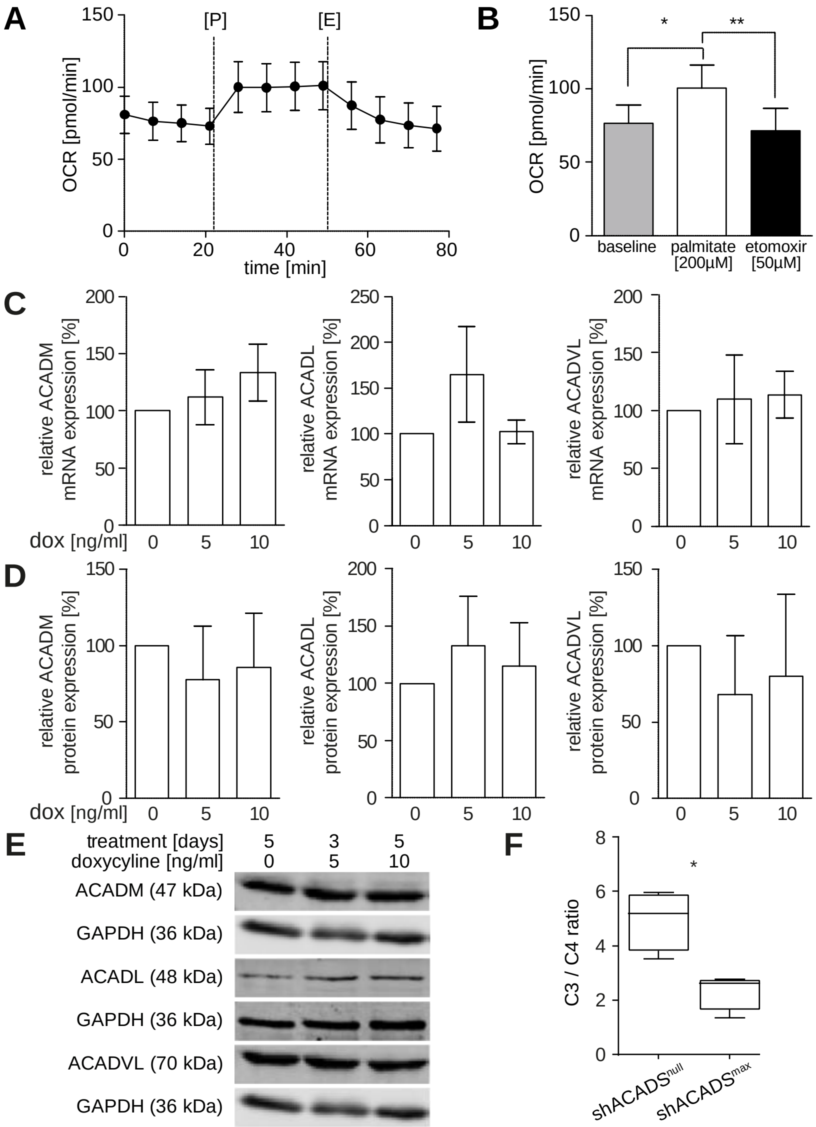

S1 Fig. Characterisation of the Huh7 cell model. (A-B) Increase of oxygen consumption after palmitic acid and inhibition by the FAO inhibitor etomoxir shows the validity of the Huh7 cell model to assess FAO. Change in oxidative consumption rate OCR (pmol/min) after stimulation and inhibition of FAO in Huh7 cells. (A) Extracellular O_2 measurement in Huh7 cells after 24h starvation in assay medium, injection of palmitic acid (200 µM) [P] and injection of CPT-1 inhibitor etomoxir (50 µM) [E]. Values of eight parallel measurements are expressed as mean ± SD. (B) Maximum increase of baseline OCR after palmitic acid injection and maximal decrease of OCR after etomoxir injection. Values of eight parallel measurements are expressed as mean + SD. ** = $p < 0.01$; * = $p < 0.05$; (Repeated measures ANOVA (Friedman test)). OCR = oxidative consumption rate. (C-E) Doxycycline-induced knockdown of ACADS does not affect mRNA expression levels or protein abundance of medium and long chain acyl-CoA dehydrogenases *ACADM*, *ACADL* and *ACADVL* in shACADS knockdown Huh7. (C) RT-qPCR analysed *ACADM*, *ACADL* and *ACADVL* mRNA of four independent experiments, shown as mean ± SD. (D-E) Western blot analysis of ACADM, ACADL and ACADVL proteins in shACADS knockdown Huh7 cell lysates. Western blotting analysed 10 µg of cell lysate per sample depicted exemplary for one (E) of four experiments (D). mRNA (C) and Protein (D-E) were harvested after 3 or 5 days of treatment with 0, 5, and 10 ng/mL doxycycline, respectively. One-sample t-test for (C) and (D) revealed no significant effect. (F) Decrease of intracellular C3/C4-acylcarnitine ratio in $shACADS^{max}$ Huh7 cells. Intracellular C3- and C4-acylcarnitine measurement in doxycycline-induced Huh7 $shACADS^{null}$ and $shACADS^{max}$ cells (treated with 0 and 10 ng/mL doxycycline, respectively). Values of four independent experiments are expressed as box plots (Boxes extend from first quartile to third quartile; median is indicated as a horizontal line; whiskers are drawn equal to 1.5 times the interquartile distance). * = $p < 0.05$; two-tailed unpaired t-test.
