# Supplementary material for: Dynamic modelling of an ACADS genotype in fatty acid oxidation – Application of cellular models for the analysis of common genetic variants
Source: PLoS One. 2019 May 23;14(5):e0216110. doi: 10.1371/journal.pone.0216110 (PMC6532850; doi:10.1371/journal.pone.0216110)
Supplement: S2 Fig — Palmitic acid was added to induce fatty acid oxidation in shACADS knockdown cells. shACADSnull, shACADSmed and shACADSmax cells treated with 0, 5 and 10 ng/mL doxycycline (dox), respectively, for shRNA induction. Intracellular acylcarnitines, assumed to represent acyl-CoAs with corresponding chain length, were extracted and measured before palmitic acid loading and after 7, 14, 21 and 28 min. Values of four independent experiments are expressed as mean ± SD (original data of single measurements are given in S1A Table). ND: concentration not measured. Time point specific comparison between shACADSnull and shACADSmax using t-test with **p < 0.01, *p < 0.05. (PDF) [file pone.0216110.s002.pdf]

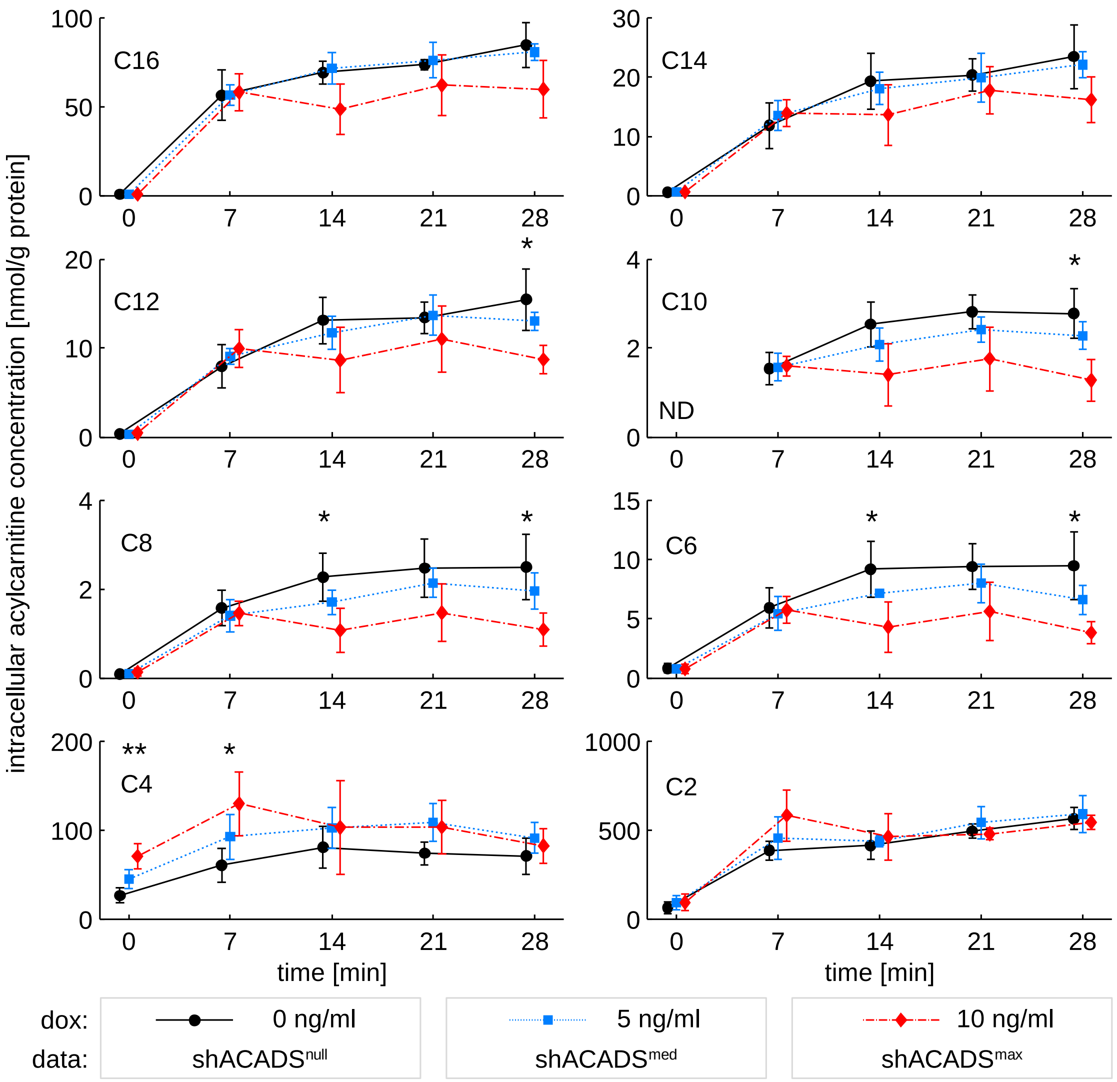

S2 Fig. Time courses of intracellular acylcarnitines after palmitic acid loading in shACADS Huh7 cells. Palmitic acid was added to induce fatty acid oxidation in shACADS knockdown cells. $shACADS^{null}$, $shACADS^{med}$ and $shACADS^{max}$ cells treated with 0, 5 and 10 ng/mL doxycycline (dox), respectively, for shRNA induction. Intracellular acylcarnitines, assumed to represent acyl-CoAs with corresponding chain length, were extracted and measured before palmitic acid loading and after 7, 14, 21 and 28 min. Values of four independent experiments are expressed as mean ± SD (original data of single measurements are given in S1A Table). ND: concentration not measured. Time point specific comparison between $shACADS^{null}$ and $shACADS^{max}$ using t-test with $^{**}p < 0.01$, $^{*}p < 0.05$.
