# Supplementary material for: Dynamic modelling of an ACADS genotype in fatty acid oxidation – Application of cellular models for the analysis of common genetic variants
Source: PLoS One. 2019 May 23;14(5):e0216110. doi: 10.1371/journal.pone.0216110 (PMC6532850; doi:10.1371/journal.pone.0216110)
Supplement: S3 Fig — Palmitic acid was added to induce fatty acid oxidation in cells transduced with a non-target shRNA. shNTCnull, shNTCmed and shNTCmax cells treated with 0, 5 and 10 ng/mL doxycycline (dox), respectively, for shRNA induction. Intracellular acylcarnitines, assumed to represent acyl-CoAs with corresponding chain length, were extracted and measured before palmitic acid loading and after 7, 14, 21 and 28 min. Values of four independent experiments are expressed as mean ± SD (original data of single measurements are given in S1B Table). ND: concentration not measured. Time point specific comparison between shNTCnull and shNTCmax using t-test with *p < 0.05. (PDF) [file pone.0216110.s003.pdf]

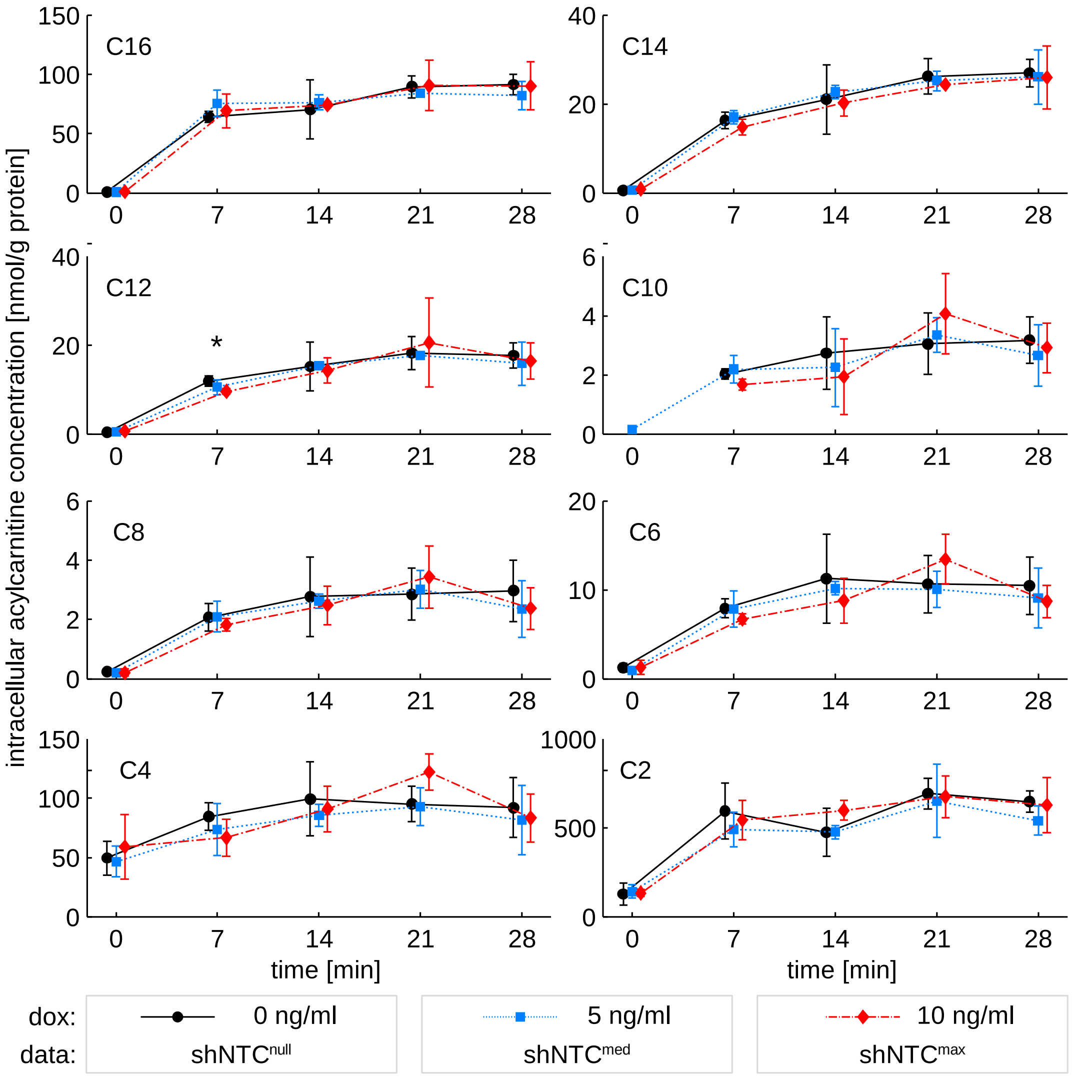

S3 Fig. Time courses of intracellular acylcarnitines after palmitic acid loading in Huh7 cells with non-target shRNA. Palmitic acid was added to induce fatty acid oxidation in cells transduced with a non-target shRNA. $shNTC^{null}$, $shNTC^{med}$ and $shNTC^{max}$ cells treated with 0, 5 and 10 ng/mL doxycycline (dox), respectively, for shRNA induction. Intracellular acylcarnitines, assumed to represent acyl-CoAs with corresponding chain length, were extracted and measured before palmitic acid loading and after 7, 14, 21 and 28 min. Values of four independent experiments are expressed as mean ± SD (original data of single measurements are given in S1B Table). ND: concentration not measured. Time point specific comparison between $shNTC^{null}$ and $shNTC^{max}$ using t-test with $^{*}p < 0.05$.
