# Supplementary material for: Dynamic modelling of an ACADS genotype in fatty acid oxidation – Application of cellular models for the analysis of common genetic variants
Source: PLoS One. 2019 May 23;14(5):e0216110. doi: 10.1371/journal.pone.0216110 (PMC6532850; doi:10.1371/journal.pone.0216110)
Supplement: S4 Fig — In each FAO reaction step of palmitic acid loaded Huh7 cells the carbon chain is shortened and C2 is produced. Fundamental chain and influx reactions for C16, C14-, C8- and C4-acylcarnitine are described by reaction rates (k16,…,k2,kinput,k14in,k8in,k4in). For a detailed description of the model and the data-driven selection of influx reactions see methods section and S1 Text. (PDF) [file pone.0216110.s004.pdf]

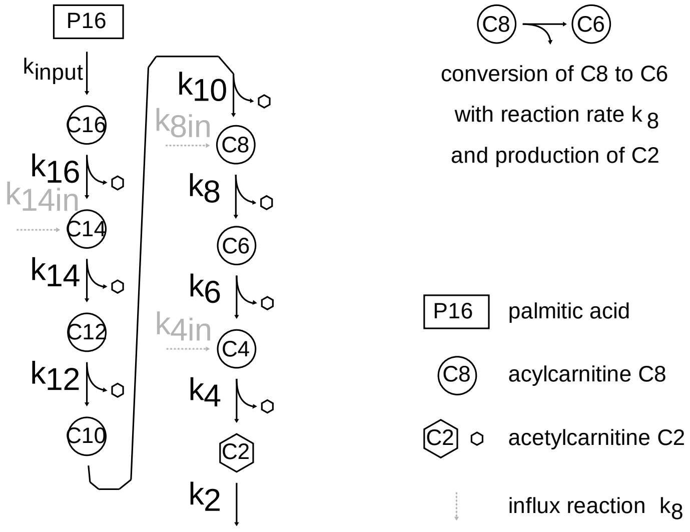

S4 Fig. Illustration of the mathematical fatty acid oxidation chain model. In each FAO reaction step of palmitic acid loaded Huh7 cells the carbon chain is shortened and C2 is produced. Fundamental chain and influx reactions for C16, C14-, C8- and C4-acylcarnitine are described by reaction rates ($k_{16}, \ldots, k_2, k_{input}, k_{14in,} k_{8in,} k_{4in}$). For a detailed description of the model and the data-driven selection of influx reactions see methods section and S1 Text.
