# Supplementary material for: Dynamic modelling of an ACADS genotype in fatty acid oxidation – Application of cellular models for the analysis of common genetic variants
Source: PLoS One. 2019 May 23;14(5):e0216110. doi: 10.1371/journal.pone.0216110 (PMC6532850; doi:10.1371/journal.pone.0216110)
Supplement: S5 Fig — Results from fitting the linear fatty acid oxidation model to the null (shACADSnull) and maximal (shACADSmax) ACADS knockdown data. Intracellular acylcarnitine levels, representing acyl-CoAs with corresponding chain length, were extracted and measured before palmitic acid loading and 7, 14, 21 and 28 min after loading in shACADS knockdown Huh7 cells, i.e. shACADSnull and shACADSmax cells treated with 0 and 10 ng/ml doxycycline (dox), respectively, for shRNA induction. Values of four independent experiments are shown as mean ± SD (original data of single measurements are given in S1A Table). # indicates significant difference (p < 0.05) between reaction rate kshACADSmax and kshACADSnull of the respective knockdown experiments (see also Fig 3C). (PDF) [file pone.0216110.s005.pdf]

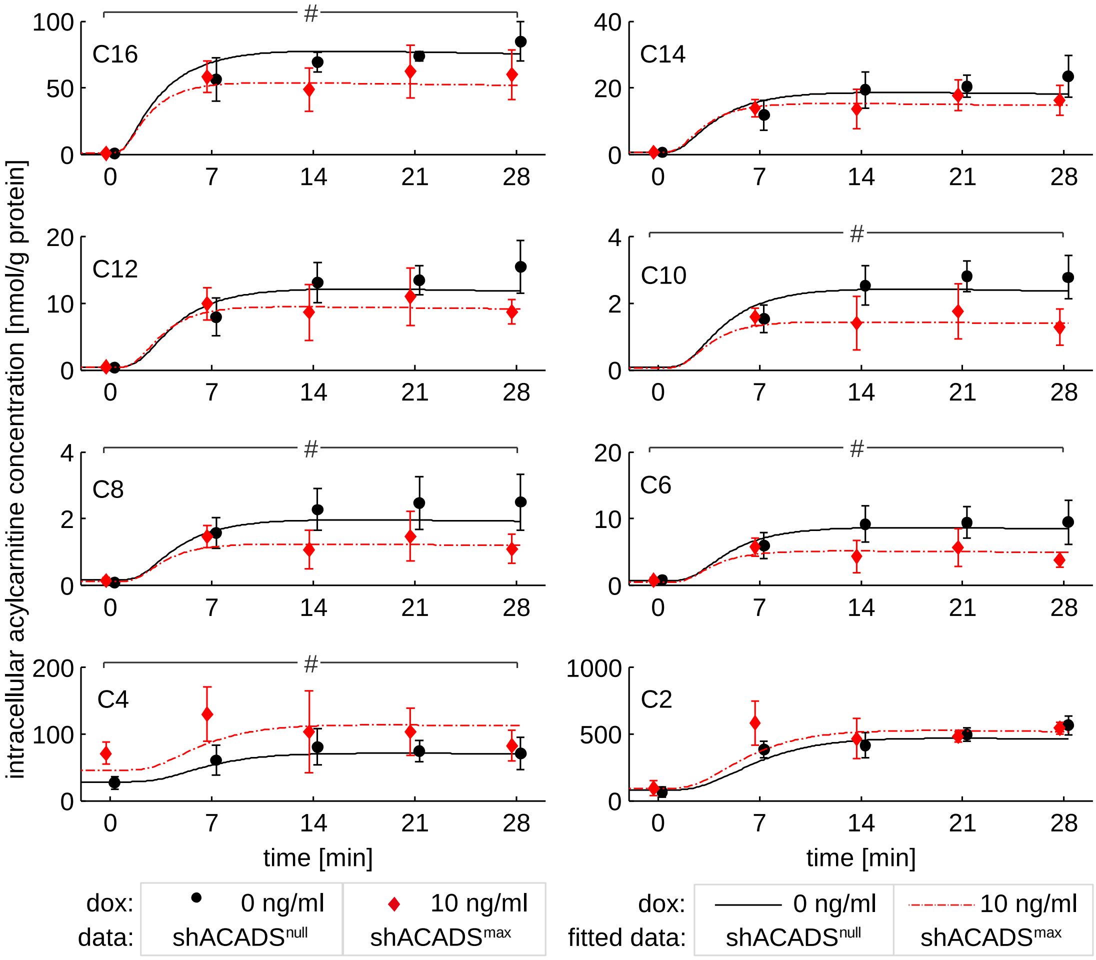

S5 Fig. Model-based analysis of intracellular acylcarnitine time course data in cells with maximal ACADS knockdown. Results from fitting the linear fatty acid oxidation model to the null ($shACADS^{null}$) and maximal ($shACADS^{max}$) ACADS knockdown data. Intracellular acylcarnitine levels, representing acyl-CoAs with corresponding chain length, were extracted and measured before palmitic acid loading and 7, 14, 21 and 28 min after loading in shACADS knockdown Huh7 cells, i.e. $shACADS^{null}$ and $shACADS^{max}$ cells treated with 0 and 10 ng/ml doxycycline (dox), respectively, for shRNA induction. Values of four independent experiments are shown as mean ± SD (original data of single measurements are given in S1A Table). # indicates significant difference ($p < 0.05$) between reaction rate $k^{shACADSmax}$ and $k^{shACADSnull}$ of the respective knockdown experiments (see also Fig 3C).
