# Supplementary material for: Dynamic modelling of an ACADS genotype in fatty acid oxidation – Application of cellular models for the analysis of common genetic variants
Source: PLoS One. 2019 May 23;14(5):e0216110. doi: 10.1371/journal.pone.0216110 (PMC6532850; doi:10.1371/journal.pone.0216110)
Supplement: S6 Fig — (A) Results from fitting the linear fatty acid oxidation model to the null (shACADSnull) and intermediate (shACADSmed) ACADS knockdown data. Intracellular acylcarnitine levels, representing acyl-CoAs with corresponding chain length, were extracted and measured before palmitic acid loading and 7, 14, 21 and 28 min after loading in shACADS knockdown Huh7 cells, i.e. shACADSnull and shACADSmed cells treated with 0 and 5 ng/ml doxycycline (dox), respectively, for shRNA induction. Values of four independent experiments are shown as mean ± SD (original data of single measurements are given in S1A Table). (B) FAO model-based quantification of differences in acylcarnitine flux dynamics. shACADSmed and shACADSnull are compared by the ratio α = kshACADSmed / kshACADSnull, reaction rates k are derived from the FAO model fits. α-values from best model fits are represented as dots with respective 95% confidence intervals. (A+B) # indicates significant difference (p < 0.05) between reaction rate kshACADSmed and kshACADSnull of the respective knockdown experiments. For details of dynamical FAO modelling, see materials and methods, S4–S6 Figs and S1 Text. Compared to the null knockdown, in the intermediate knockdown reaction rate k4 is significantly decreased, whereas reaction rate k8 is significantly increased. (PDF) [file pone.0216110.s006.pdf]

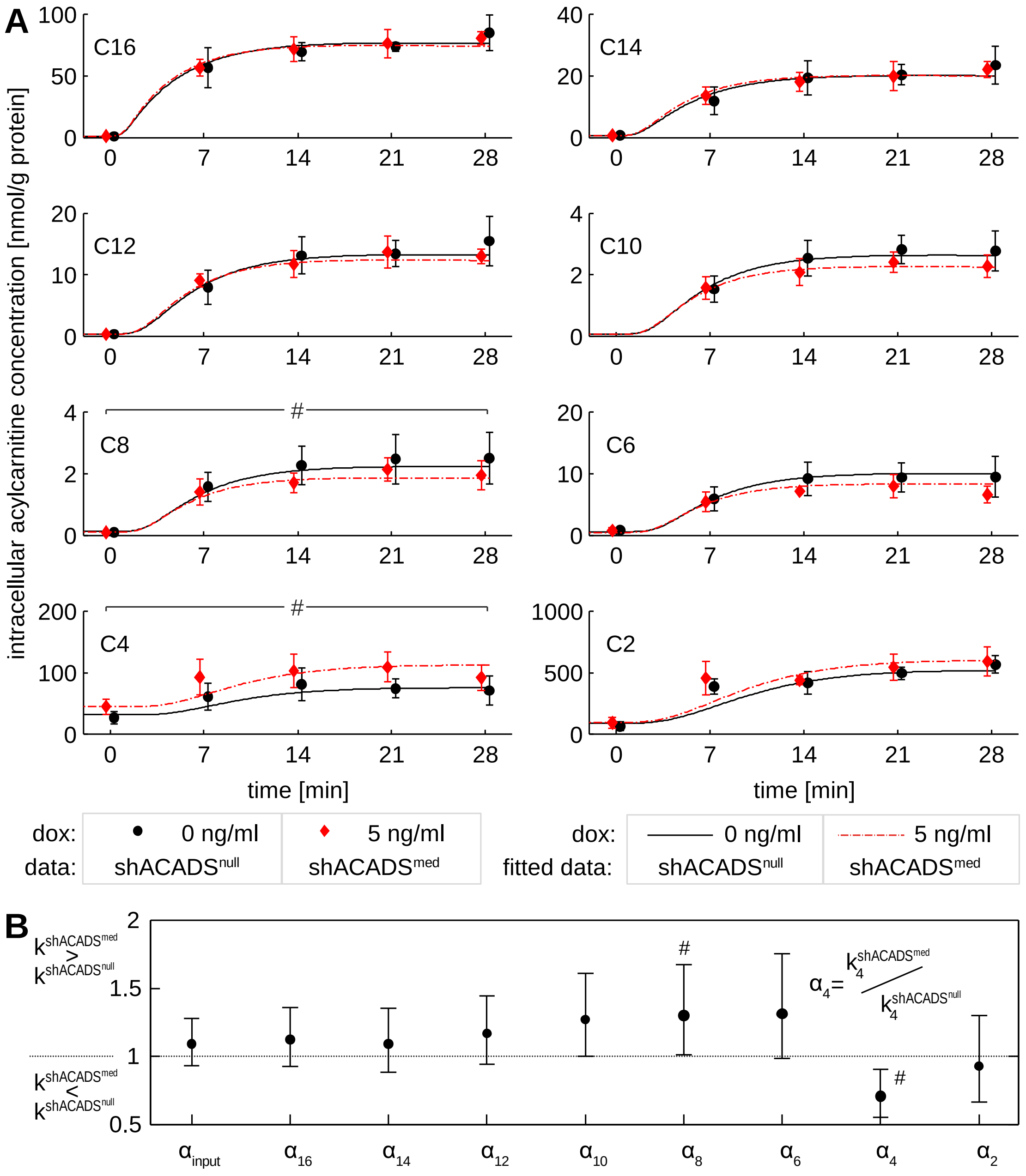

S6 Fig. Model-based analysis of intracellular acylcarnitine time course data in cells with intermediate ACADS knockdown. (A) Results from fitting the linear fatty acid oxidation model to the null ($shACADS^{null}$) and intermediate ($shACADS^{med}$) ACADS knockdown data. Intracellular acylcarnitine levels, representing acyl-CoAs with corresponding chain length, were extracted and measured before palmitic acid loading and 7, 14, 21 and 28 min after loading in shACADS knockdown Huh7 cells, i.e. $shACADS^{null}$ and $shACADS^{med}$ cells treated with 0 and 5 ng/ml doxycycline (dox), respectively, for shRNA induction. Values of four independent experiments are shown as mean ± SD (original data of single measurements are given in S1A Table). (B) FAO model-based quantification of differences in acylcarnitine flux dynamics. $shACADS^{med}$ and $shACADS^{null}$ are compared by the ratio $\alpha = k^{shACADSmed} / k^{shACADSnull}$, reaction rates k are derived from the FAO model fits. α-values from best model fits are represented as dots with respective 95% confidence intervals. (A+B) # indicates significant difference ($p < 0.05$) between reaction rate $k^{shACADSmed}$ and $k^{shACADSnull}$ of the respective knockdown experiments. For details of dynamical FAO modelling, see materials and methods, S4-6 Figs and S1 Text. Compared to the null knockdown, in the intermediate knockdown reaction rate k_4 is significantly decreased, whereas reaction rate k_8 is significantly increased.
