# Supplementary material for: Dynamic modelling of an ACADS genotype in fatty acid oxidation – Application of cellular models for the analysis of common genetic variants
Source: PLoS One. 2019 May 23;14(5):e0216110. doi: 10.1371/journal.pone.0216110 (PMC6532850; doi:10.1371/journal.pone.0216110)
Supplement: S1 Text — (PDF) [file pone.0216110.s007.pdf]

S1 Text. Description of the fatty acid oxidation model.

The FAO model was described as a linear cascade of subsequent, irreversible first-order reactions using ordinary differential equations (ODEs) with mass action kinetics (see T1 Fig). Measured intracellular acylcarnitine concentrations were used as a reflection for acyl-CoA concentrations, described as observables $C_{16}, C_{14}, \dots, C_2$ in the model. Since only few intermediate products of a FAO cycle were measured, we combined the four FAO cycle reaction steps, i.e. dehydrogenation, hydration, oxidation, thiolysis, into one fundamental reaction step (e.g. $C_6 \overset{k_6}{\rightarrow} C_4 + C_2$, with the reaction rate k_6). During each cascade step the carbon chain is shortened by two carbon atoms. We also took into account exchange reactions between the FAO pathway and other active biochemical pathways by adding acylcarnitine-specific influx (e.g. $\xrightarrow{k_{4in}} C_4$) and outflux (e.g. $C_4 \xrightarrow{k_{4out}}$). The concentration change of each observable over time was modelled by systems of ODEs (e.g. $\frac{dC_4}{dt} = k_{4in} - k_{4out}C_4 + k_6C_6 - k_4C_4$). The change of C_4 thus depends on the concentrations of C_4 and C_6, the reaction rates of the influx and outflux reactions (k_{4in}, k_{4out}), and the rates (k_6, k_4) of conversion reactions $C_6 \overset{k_6}{\rightarrow} C_4 + C_2$ and $C_4 \overset{k_4}{\rightarrow} C_2 + C_2$.

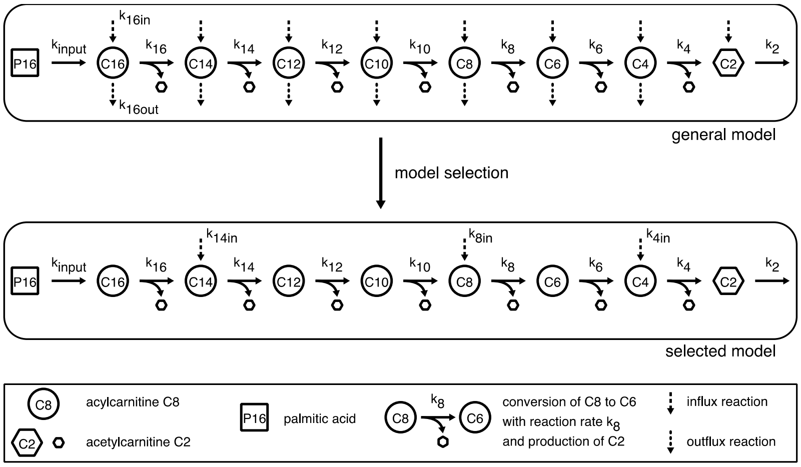

T1 Fig: Schematic representation of the fatty acid oxidation chain model. The general model includes influx and outflux reactions for each compound. After data-driven model selection the selected model contains only influx reactions for C14, C8 and C4.

The ODE system of the general fatty acid oxidation model (see T1 Fig) reads

$$
\begin{aligned}
\frac{P16}{dt} &= \text{P16loading} - k_{input} \cdot P16 \\
\frac{C_{16}}{dt} &= k_{16in} - k_{16out} \cdot C_{16} + k_{input} \cdot P16 - k_{16} \cdot C_{16} \\
\frac{C_{14}}{dt} &= k_{14in} - k_{14out} \cdot C_{14} + k_{16} \cdot C_{16} - k_{14} \cdot C_{14} \\
\frac{C_{12}}{dt} &= k_{12in} - k_{12out} \cdot C_{12} + k_{14} \cdot C_{14} - k_{12} \cdot C_{12} \\
\frac{C_{10}}{dt} &= k_{10in} - k_{10out} \cdot C_{10} + k_{12} \cdot C_{12} - k_{10} \cdot C_{10} \\
\frac{C_8}{dt} &= k_{8in} - k_{8out} \cdot C_8 + k_{10} \cdot C_{10} - k_8 \cdot C_8 \\
\frac{C_6}{dt} &= k_{6in} - k_{6out} \cdot C_6 + k_8 \cdot C_8 - k_6 \cdot C_6 \\
\frac{C_4}{dt} &= k_{4in} - k_{4out} \cdot C_4 + k_6 \cdot C_6 - k_4 \cdot C_4 \\
\frac{C_2}{dt} &= k_{2in} + 2k_4 \cdot C_4 + k_6 \cdot C_6 + k_8 \cdot C_8 + k_{10} \cdot C_{10} + k_{12} \cdot C_{12} + \\
&\quad + k_{14} \cdot C_{14} + k_{16} \cdot C_{16} - k_2 \cdot C_2
\end{aligned}
$$

Equation (1)

The initial condition for each compound is described by additional parameters ($C_{16init}, C_{14init}, \ldots, C_{2init}$). Loading of Huh7 cells with palmitic acid is represented using a scaled normal distribution curve as input function for P16. This means that the state variable of P16 changes its value by a certain amount at a specific timepoint *t* during the simulation. We chose as input function:

$$\text{P16loading} = \text{P16total} \cdot \frac{1}{\sqrt{2\pi d^2}} \exp\left\{-\frac{(t-tp)^2}{2d^2}\right\}$$

with P16total being the total palmitic acid amount (i.e. area under the curve, here set to 10000 [nmol / g protein]), *tp* the time point of palmitic acid loading (at time point 1 [min]) and *d* being the duration of palmitic acid loading (set to 0.5 [min]). In order to quantify differences in cascade reaction rates between two experimental conditions (e.g. between $\text{shACADS}^{\text{null}}$ and $\text{shACADS}^{\text{max}}$) we used two different FAO models. The ODE system of the first model (M1) is represented as in Equation (1). For the second model (M2) we introduced for all cascade reactions rates ($k_{input}, k_{16}, k_{14}, \ldots, k_2$) condition specific prefactors ($\alpha_{input}, \alpha_{16}, \alpha_{14}, \ldots, \alpha_2$). As an example, the change of C4 over time is described in the first model M1 (e.g. $\text{shACADS}^{\text{null}}$) by

$$\frac{C_4^{M1}}{dt} = k_{4in} - k_{4out} \cdot C_4 + k_6 \cdot C_6 - k_4 \cdot C_4$$

and in the second model M2 (e.g. $\text{shACADS}^{\text{max}}$)

$$\frac{C_4^{M2}}{dt} = k_{4in} - k_{4out} \cdot C_4 + \alpha_6 \cdot k_6 \cdot C_6 - \alpha_4 \cdot k_4 \cdot C_4$$

with initial conditions C_{4init}^{M1} and C_{4init}^{M2}. An α_4-value of 1 then denotes that the reaction rate between the two models is not different. Note that the influx and outflux rates k_{4in} and k_{4out} are the same for both models, assuming that influx and outflux reactions should be independent of the knockdown. The model simulations were compared to the time course acylcarnitine data on log10-scale obtained from the knockdown experiments. This comparison was performed on log10-scale to account for log-normally distributed measurement noise. We used maximum likelihood estimation to obtain model parameters which optimally describe the measured data. The parameter fitting was carried out on log10-scale to ensure efficient estimates for values being potentially different by orders of magnitude. A profile likelihood approach was used to check for parameter identifiability and to compute 95% confidence intervals for parameter values [1].

For reducing the model complexity, we performed a model selection for all influx and outflux reactions. To this end all models with all possible influx and outflux combinations were fitted individually to the control ($\text{shACADS}^{\text{null}}$) and knockdown ($\text{shACADS}^{\text{max}}$) data using model M1 and M2. Yet adding additional parameters to the model will increase the model fit and might result in over fitting. Therefore, we used the Bayesian information criterion (BIC), which includes a penalty term for the number of model parameters:

$$BIC = -2 \cdot \log(\hat{L}) + k \cdot \log(n)$$

Here $\hat{L}$ is the maximised likelihood of the model, k the number of model parameters and n the amount of measured data points. A lower BIC value results from either fewer parameters, better data fitting or both. T2 Fig shows the result for the best 10 models. The selected model with the lowest Bayesian information criterion (BIC) score only

contains influx reactions for C14, C8 and C4 and no outflux out of the system except for the reaction $C_2 \xrightarrow{k_2}$ with reaction rate k_2 (see T1 Fig). This means all other influx and outflux reaction rates are set to 0, the ODE system in equation (1) reduces accordingly.

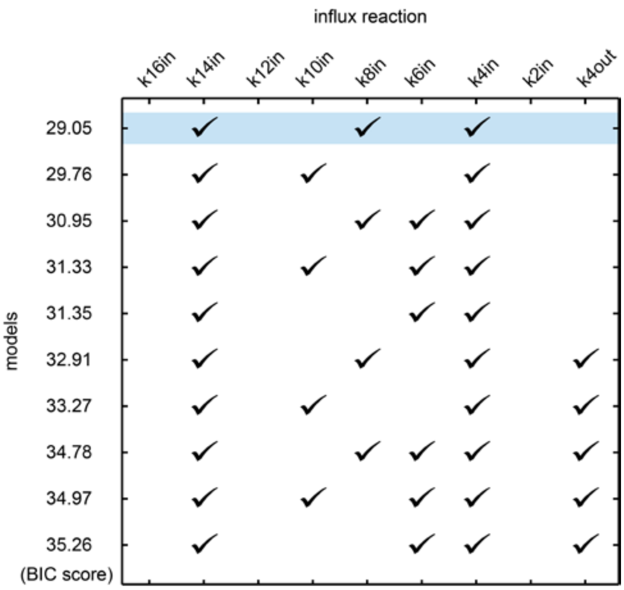

T2 Fig: Comparison of the 10 best models according to Bayesian information criterion (BIC) score. Each row correspond to a single model. Influx reactions present in the respective model are denoted by ticks. Only the outflux parameter of C4 is shown as none of the other outflux parameters were present in the best 10 models. The model in the first row (blue shaded area) with the lowest BIC score was chosen for further analysis.

Reference

1. Raue A, Kreutz T, Maiwald T, Bachmann J, Schilling M, Klingmüller U, Timmer J. Structural and practical identifiability analysis of partially observed dynamical models by exploiting the profile likelihood. Bioinformatics. 2009; 25: 1923–1929. DOI: 10.1093/bioinformatics/btp358.
